# Is serum fibroblast growth factor 21 associated with the severity or presence of coronary artery disease?

**DOI:** 10.5937/jomb0-30191

**Published:** 2022-04-08

**Authors:** Gokay Nar, Sanlialp Sara Cetin, Rukiye Nar, Oguz Kilic, Ozen Mehmet Furkan, Guven Gunver, Sevgican Cihan Ilyas

**Affiliations:** 1 Pamukkale University, Faculty of Medicine, Department of Cardiology, Denizli, Turkey; 2 Servergazi State Hospital, Department of Cardiology, Denizli, Turkey; 3 Pamukkale University, Faculty of Medicine, Department of Medical Biochemistry, Denizli, Turkey; 4 Ismail Karakuyu State Hospital, Department of Cardiology, Kutahya, Turkey; 5 Istanbul Medical Faculty, Department of Biostatistics, Istanbul, Turkey

**Keywords:** FGF21, stable angina pectoris, coronary artery disease, FGF21, stabilna angina pektoris, koronarno arterijsko oboljenje

## Abstract

**Background:**

Recent studies have shown that increased circulating concentrations of fibroblast growth factor 21 (FGF21) are associated with obesity, metabolic disorder, and atherosclerosis. However the relationship between FGF21 and coronary artery disease (CAD) is controversial This study was planned to investigate the role of FGF21 in CAD development and CAD severity.

**Methods:**

Seventy-eight patients with stable angina pectoris (SAP) (lesion positive) and 40 control patients (lesion negative) with similar cardiovascular risk factors were included in the study. Serum FGF21 levels were measured by ELISA method. CAD severity was evaluated by using SYNTAX and GENSINI risk scores.

**Results:**

FGF21 concentrations were found significantly higher in the SAP group than in the control group. [101.18 ± 141.62 vs. 47.93 ± 58.74 pg/mL; p = 0.03], no correlation was found between the SYNTAX (r = 0.146 and p = 0.134) and GENSINI (r = 0.211 and p = 0.084) scores with serum FGF21 levels. There was a negative relationship between serum FGF21 and serum HDL-C levels in correlation analysis (r = - 0.272; p = 0.026).

**Conclusions:**

The serum FGF21 levels are different between SAP and control patients. FGF21 is a marker for CAD diagnosis, but not for the evaluation of CAD severity.

## Abstract (sr)

**Uvod:**

Poslednja istraživanja su pokazala da je pove}anje koncentracija u cirkulaciji fibroblastnog faktora rasta 21 (FGF21) praćeno sa gojaznošću, metaboličkim poremećajem i aterosklerzom. Međutim, odnos između FGF21 i koro narnog arterijskog oboljenja (KAO) je kontraverzan. U ovom izučavanju je planirano da se ispita uloga FGF21 u razvoju KAO kao i težina samog oboljenja.

**Metode:**

U izučavanje je uključeno sedamdeset osam pacijenata sa stabilnom anginom pektoris (SAP) (pozitivna lezija) i 40 kontrolnih pacijenata (lezija negativna) sa sličnim faktorima rizika. Nivoi serumskog FGF21 mereni su ELISA me todom. Težina KAO procenjivana je SYNTAX i GENSI faktorima rizika.

**Rezultati:**

Koncentracije FGF21 bile su značajno vi{e u grupi sa SAP nego u kontrolnoj grupi. [101,18 ± 141,62 vs. 47,93 ± 58,74 pg/mL; p = 0,03]. Nije utvrđena korelacija između SYNTAX (r = 0,146 i p = 0,134) i GENSI (r = 0,211 i p = 9,084) skorova sa nivoima serumskog FGF21. Nađen je negativan odnos između serumskog FGF21 i nivoa HDL-C u serumu primenom korelacione analize (r = - 0,272; p = 0,026).

**Zaključak:**

Nivoi FGF21 u serumu su različiti kod SAP i kontrolnih pacijenata. FGF21 je marker za dijagnostikovanje KAO, ali ne i za procenu težine KAO.

## Introduction

Cardiovascular diseases (CVDs) are among the most common causes of morbidity and mortality [1]. Despite the developments in evidence-based medical treatments and revascularisation strategies, CVD still continues to be a global health problem and leads to a significant burden on the health system [2] [3]. Atherosclerosis has been assumed to be a chronic inflammatory process, and the immune system's response to oxidised lipoproteins, in particular, initiates this process [4] [5]. Many inflammatory biomarkers, especially cytokines, have been investigated for the prevention and early detection of CVD [2] [6].

Adipose tissue secretes many bioactive adipokines. These adipokines not only affect the metabolism, but also have many effects on the cardiovascular system [3]. Fibroblast growth factor (FGF) is a hormone-like structure that plays a role in cell proliferation, hyperplasia, differentiation, and angiogenesis [7]. FGF21 is a member of the FGF endocrine subfamily.

High serum FGF21 levels have been shown in obese patients with type 2 diabetes mellitus and metabolic syndrome [8]. Although the relationship between FGF21 and CVD has been shown in some studies, its role in the pathophysiology of CAD is still not fully understood [9]. We aimed to investigate the role of FGF21 in CAD development and CAD severity in patients with SAP.

## Materials and Method

### Patient population

In this cross-sectional case-control study, the patients who underwent coronary angiography with suspicion of CAD at the Pamukkale University Cardiology Department between January 1, 2018 and January 5, 2018 were included. All patients were evaluated in terms of age, gender, history, family history, smoking, presence of comorbidities, and drug use before coronary angiography. The physical examinations were performed. A comprehensive, twodimensional transthoracic echocardiography (2D-TTE) examination (GE vivid S5) was performed for all patients. The left ventricular ejection fraction (LVEF) was calculated by the bi-planar Simpson method.

Previous acute coronary syndrome (ACS), documented CAD, chronic or inflammatory systemic diseases, autoimmune diseases, malignancies, severe heart valve diseases, a left ventricular ejection fraction <50%, chronic kidney failure [Cockcroft-Gault formula calculated glomerular filtration rate 90 mL/min1.73 m^2^)], haematological disorders, thyroid dysfunctions, pregnancy, and suspicious pericarditis or myocarditis were determined as exclusion criteria. Stable angina pectoris (SAP) was defined as the presence of typical chest pain or equivalent symptoms during exercise and a positive treadmill test or visualisation of ischaemia on stress echocardiography and myocardial perfusion scintigraphy. Patients who did not have any atherosclerotic lesions on coronary angiography were included in the control group. This study was carried out with the approval of the Institutional Review Board of the Pamukkale University Medical Faculty, and informed consent was obtained from all registered patients.

### Blood samples

Peripheral venous blood samples of the patients were collected after eight to 12 hours of fasting. Fasting serum glucose (FSG), creatinine, triglyceride (TG), total cholesterol (TC), low density lipoprotein cholesterol (LDL-C), high density lipoprotein cholesterol (HDL-C) and C-reactive protein (CRP) levels were analysed with the electrochemiluminescence method on a Cobas 702 autoanalyser (Roche Diagnostics GmbH, Mannheim, Germany). The hemogram parameters were analysed with the Mindray BC-6800 system through the electrical impedance method.

### FGF21 level measurement

Blood samples were centrifuged at 3,000 x g for 10 minutes, and the serum was stored at -80 degrees Celsius (°C) until the analysis. Serum FGF21 concentrations were measured in accordance with the manufacturer's protocol with a commercial ELISA kit (YLA0238HU, Shanghai YL Biotech Co., Ltd). The concentration range of the assay was 5 to 1,500 pg/mL. The absorbance was measured at 450 nm with a Biotek Elx800 microplate reader (BioTek Instruments Inc., U.S.A.). The data were processed with the Gen5 Data Analysis software (BioTek Instruments Inc., U.S.A.). The variation coefficients within and between experiments were determined as < (8%) and < (12%), respectively.

### Coronary angiography

Coronary angiography was performed using standard technique (GE Innova 2100) at the Pamukkale University Cardiology Department Selective cine angiographic images of the coronaries were recorded with a digital angiographic system. The significant CAD was defined as narrowing of the vessel lumen diameter > 50%, including the three major coronary arteries and the first branches of the left anterior descending artery or circumflex artery. Diagnostic angiograms were recorded using a digital media viewer and their analysis was randomly performed by two experienced cardiologists, blinded to the study protocol.

### Angiographic risk scoring

Atherosclerotic lesion severity was evaluated by using SYNTAX and GENSINI scores. SYNTAX score is a scoring system developed based on the number, location, function and complexity of the coronary lesions, calculated for stenosis diameter of 50% or greater in vessels of 1.5 mm or more in diameter. The final online updated version [2.11] was used (www.syntaxscore.com) [10]. In the GENSINI scoring system, the lesions were classified as 0-25%, 26-50%, 51-75%, 76-90% according to the degree of angiographic stenosis, and were scored 1,2,4,8,16 and 32 points, respectively. Then the score was multiplied by the coefficient defined according to the localization of the lesion. (Left main coronary artery, 5; proximal segment of left anterior descending (LAD) coronary artery or left circumflex (LCx) artery, 2.5; middle segment of LAD or LCx coronary artery, 1.5; distal segment of LAD and LCx, first diagonal branch, first marginal branch, right coronary artery, posterior descending artery, 1; and intermediate artery and second diagonal and second large marginal branches, 0.5) [11].

### Statistical analysis

Statistical analysis were performed on SPSS version 20.0. Normality check for continuous variables were performed by Kolmogorov-Smirnov test and normality assumption was proven for variables and subgroups. Chi-square test was used for categorical variables and independent sample t test was used for continuous variables. Pearson correlation was used to determine relationship between continuous variables. Statistical significance level (alpha) was determined as 0.05.

## Results

A total of 118 consecutive patients were enrolled in the study. The baseline demographic, clinical features, laboratory test values and other parameters of the groups are shown in Table 1. The patients in the SAP group were significantly older than those in the control group (63.29 ± 10.79 vs 58.88 ± 11.49; p = 0.04), and there were more male patients in the SAP group (74% vs. 58%; p =0.015). The incidences of cardiovascular risk factors, such as hypertension, diabetes and smoking, were similar in both groups (p> 0.05). LVEF was different between the SAP and control groups (53.45 ± 9.16% vs. 57.03 ± 6.23%, respectively; p = 0.03), and the SAP group had lower serum HDL-C levels (41.57 ± 11.40 vs. 54.90 ± 34.47; p = 0.02).

**Table 1:** Baseline demographics – clinical characteristics, laboratory and angiographic parameters. Abbreviations: FSG, fasting serum glucose; WBC, white blood cells; TChol, total cholesterol; LDL-C, low-density lipoprotein cholesterol; HDL-C, high-density lipoprotein cholesterol; TG, triglycerides; CRP, C-reactive protein; FGF21, fibroblast growth factor 21; LVEF, left ventricular ejection fraction, SAP, Stable angina pectoris.

Baseline demographics and clinicalcharacteristics	SAP group (lesion +)<br>(n=78)	Control group (lesion -)<br>(n=40)	p value
Age (y)	63.29 ± 10.79	58.88 ± 11.49	0.04
Males, n (%)	58 (74%)	21 (53%)	0.015
Hypertension, n (%)	46 (58%)	24 (60%)	0.915
Diabetes Mellitus, n (%)	29 (37%)	12 (30%)	0.438
Smoking, n (%)	17 (21%)	7 (18%)	0.583
LVEF (%)	53.45 ± 9.16	57.03 ± 6.23	0.03
Laboratory data			
FSG (mmol/L)	7.04 ± 2.73	6.64 ± 1.92	0.41
Creatinine (mmol/L)	88.4 ± 75.14	87.51 ± 27.40	0.51
Haemoglobin (g/L)	133.2 ± 19.0	135.3 ± 17.3	0.56
WBC (×10^9^/L)	9.34 ± 2.86	9.21 ± 2.89	0.82
TChol (mmol/L)	4.62 ± 1.32	4.91 ± 1.60	0.29
LDL-C (mmol/L)	2.74 ± 1.15	2.79 ± 1.29	0.85
HDL-C (mmol/L)	1.08 ± 0.30	1.42 ± 0.89	<0.00
TG (mmol/L)	1.79 ± 1.24	1.65 ± 0.99	0.54
CRP (mg/mL)	12.9 ± 27.1	5.8 ± 5.2	0.12
FGF21 (pg/mL)	101.18 ± 141.62	47.93 ± 58.74	0.03
Angiographic data			
SYNTAX score	23.80 ± 9.53		
GENSINI score	50.01 ± 32.15		

**Figure 1:**
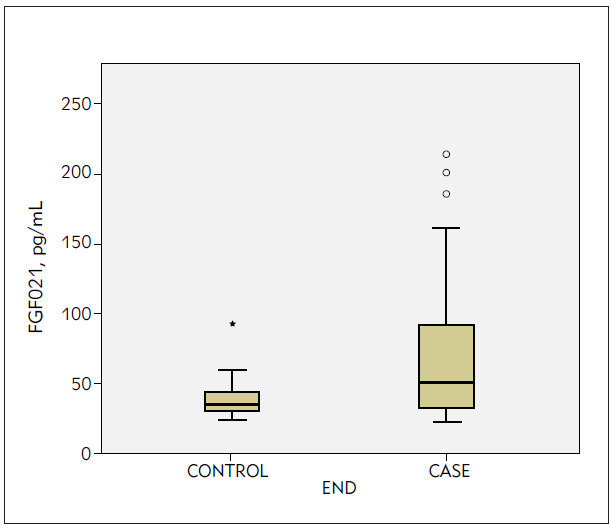
The comparison of serum FGF21 levels in both groups

**Figure 2:**
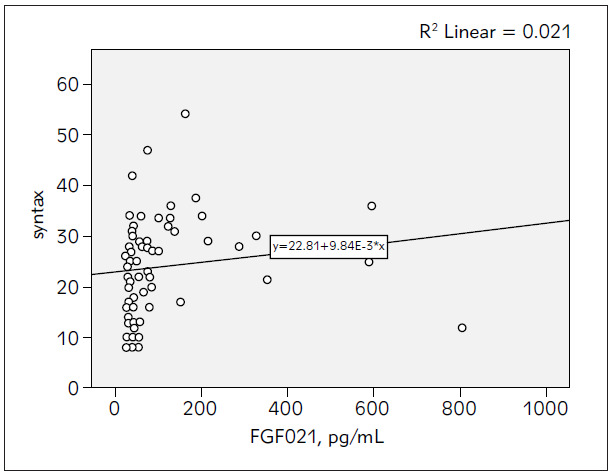
The correlation between FGF21 levels and SYNTAX score

**Figure 3:**
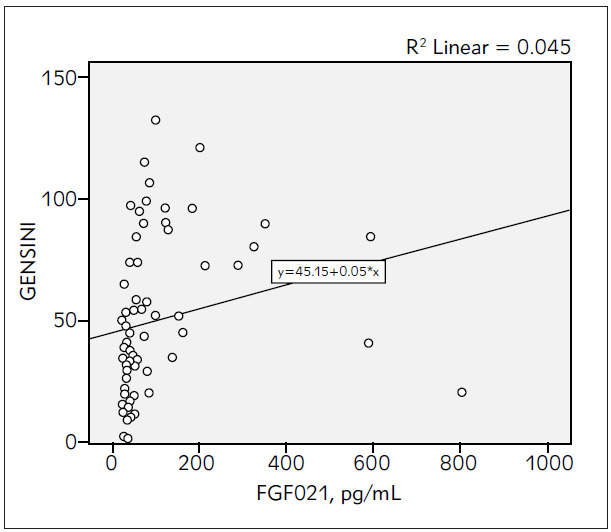
The correlation between FGF21 levels and GENSINI Risk score

Serum FGF21 levels of the SAP and control groups were measured as 101.18 ± 141.62 pg /mL and 47.93 ± 58.74 pg/mL, respectively, and showing significantly higher levels in the SAP group (p = 0.03), (Figure 1). Levels of another inflammatory biomarker, CRP, were similar in both groups (12.9 ± 27.1 vs 5.8 ± 5.2 mg/mL; p = 0.12). There was a significant and negative relationship between serum FGF21 and serum HDL-C levels based on correlation analysis (r= -0.272, p = 0.026), (Table 2). The SYNTAX and GENSINI scores of the SAP group were 23.80 ± 9.53 and 50.01 ± 32.15, respectively. However, there was no correlation between the CAD severity risk scores and serum FGF21 levels (r = 0.146, p = 0.134 and r = 0.211 and p = 0.084, respectively), (Figure 2 and Figure 3).

**Table 2:** The correlation of FGF21 levels with baseline clinical, biochemical, hemorheological and other parameters. Abbreviations: FSG, fasting serum glucose; WBC, white blood cells; TChol, total cholesterol; LDL-C, low-density lipoprotein cholesterol; HDL-C, high-density lipoprotein cholesterol; TG, triglycerides; CRP, C-reactive protein; FGF21, fibroblast growth factor 21; LVEF, left ventricular ejection fraction

FGF21	Correlation	Sig. (2-tailed)
Age	0.147	0.232
LVEF	-0.075	0.544
WBC	0.129	0.293
Hemoglobin	0.046	0.707
FSG	-0.074	0.547
Creatinin	-0.103	0.402
TChol	0.046	0.712
LDL-C	0.007	0.956
HDL-C	-0.272^*^	0.026
TG	-0.082	0.510
CRP	-0.081	0.512
SYNTAX	0.146	0.234
GENSİNİ	0.211	0.084

## Discussion

In this study, we evaluated the role of FGF21 in the diagnosis and severity of CAD. Although, we found a significant difference between the serum FGF21 levels and the diagnosis of CAD in the SAP and control groups, there was no correlation between the serum FGF21 levels and the SYNTAX and GENSINI scores.

Several studies have shown that serum FGF21 levels may increase in cardiovascular risk factors such as obesity, hypertension and type 2 diabetes. How ever, the results of human studies investigating the physiological functions of FGF21 have been inconsistent and contradictory [12] [13]. In a study involving 253 Chinese patients, high serum FGF21 levels were an independent risk factor for CAD [14]. An et al. [15] found that FGF21 levels increased in diabetic patients and high levels were associated with diabetic complications, including carotid plaques. In another study with 235 patients, the CAD group had higher serum FGF21 compared to non-CAD group [14]. Consistent with these studies, we found a statistically significant difference between the serum FGF21 levels of the SAP and control groups. However, Lee et al. [16] did not find significant differences between serum FGF21 levels in patients with or without CAD, using coronary CT angiography to diagnose CAD. The authors explained that this result was due to group matchings in terms of body mass index, age, inflammatory, and lipid markers, and low serum FGF21 levels [16]. A study that investigated the role of FGF21 in SAP found higher FGF21 levels (SAP: 323.16 ± 434.66 vs. control: 266.46 ± 417.13 pg/mL; p = 0.039), similar to our study; however, multiple regression analyses showed that FGF21 levels could not be used as a marker for SAP [17]. In another study, serum FGF21 levels were found higher in patients with unstable angina pectoris than in the SAP and control group, and there was no difference between serum FGF21 levels in SAP and control subjects un like to our study [18]. This may be due to study design differences, heterogeneities in patients' cardiovascular risk profiles, insulin resistance, drug use, body mass index, visceral fat distribution, and gender distribution.

A few studies have investigated the relationship between CAD severity and FGF21. In a study of 417 patients, the serum FGF21 levels of the patients with CAD and without CAD were similar regardless of the presence of diabetes, and there was no association between the serum FGF21 levels and the severity of CAD defined by the number of stenotic vessels and segments [19]. In Park et al. [20] study, no relationship was found between serum FGF21 levels and the severity of CAD determined by SYNTAX scores. In another study, Kim et al. [21] initially observed a significant correlation between serum FGF21 levels and GENSINI and EXTENT scores of 120 patients; however, in the final analysis, they found that there was no relationship between FGF21 levels and the risk scores in diabetic patients (r = 0.332, p = 0.055; and r = 0.296, p = 0.084, respectively). Similar to these studies, we did not find any relationship bet ween serum FGF21 levels and SYNTAX and GENSINI scores.

In this study, we found a negative correlation between FGF21 levels and only HDL-C. No relationship was observed between FGF21 and parameters such as FSG, CRP, non-HDL-C lipid values, creatinine, and LVEF. Indeed, Matuszek et al. [22] confirmed that circulating FGF21 levels had a negative correlation with HDL-C. Similar to our study, Lee et al. [16] failed to demonstrate an association of serum FGF21 levels with FSG and CRP values, and suggested that higher FGF21 levels may be a reflection of hyperinsulinemia rather than high serum glucose levels.

### Limitations of the study

The main limitations of our study include its cross-sectional design, small sample, and failure to determine long-term prognoses. The correlation analysis results may also be affected by various uncorrected confounding factors of life. The study was also limited by the collection of blood samples during the stable period before angiography and the inability to measure FGF21 levels simultaneously with the occurrence of chest pain, as acute angina may increase serum FGF21 levels.

## Conclusion

Although the serum FGF21 levels were different between patients with and without SAP in this study, there was no significant relationship between angiographic scores of CAD severity and FGF21 levels. One of the strengths of the study is that FGF21 levels, lipid profile, and coronary artery disease severity were evaluated simultaneously. However, larger studies are needed to determine the role of FGF21 in CAD development and progression.

## Dodatak

### Conflict of interest statement

The authors reported no conflict of interest regarding the publication of this article.
